# Conversion surgery for esophageal and esophagogastric junction cancer

**DOI:** 10.1007/s10147-024-02639-4

**Published:** 2024-10-22

**Authors:** Yoshiaki Shoji, Kohei Kanamori, Kazuo Koyanagi, Tetsuya Otsuka, Rie Nakashima, Kohei Tajima, Mika Ogimi, Yamato Ninomiya, Miho Yamamoto, Akihito Kazuno, Takayuki Nishi, Masaki Mori

**Affiliations:** Department of Gastroenterological Surgery, Tokai University School of Medicine, 143, Shimokasuya, Isehara-City, Kanagawa 259-1193 Japan

**Keywords:** Esophageal cancer, Esophageal squamous cell carcinoma, Esophagogastric junction cancer, Esophagogastric junction adenocarcinoma, Conversion surgery, Induction therapy

## Abstract

As a result of the recent advances in first-line treatment including chemotherapy, radiation therapy, targeted therapy, and immune checkpoint inhibitor immunotherapy (ICI) for locally advanced/metastatic initially unresectable esophageal and esophagogastric junction cancer, surgery aiming at cure after initial treatment, so-called “conversion surgery” has become more common in this field. Several studies have indicated encouraging survival outcomes for patients after conversion surgery with R0 resection. However, various issues, such the utility and the safety of conversion surgery remain unclear. In this review, we will focus on the surgical treatment for initially unresectable esophageal and esophagogastric junction cancer after first- or later- line treatment and review recent evidence regarding the safety and the efficacy of conversion surgery. Multidisciplinary treatment including surgery may serve as a novel treatment strategy for esophageal and esophagogastric junction cancer, thus provide a curative treatment option and potentially contribute to better prognosis for initially untreatable diseases.

## Introduction

Recent development of systemic therapy and radiation therapy has dramatically improved prognosis for patients with locally advanced/metastatic unresectable esophageal and esophagogastric junction cancer. Definitive chemoradiation is frequently selected for patients with locally advanced diseases, whereas the combination of chemotherapy plus targeted therapy/immune checkpoint inhibitor immunotherapy (ICI), or doublet ICI is often performed for metastatic diseases according to tumor histology and HER2 status. Although recent studies on chemotherapy + ICI or doublet ICI have reported a high objective response rate [1] [2] [3] [4], complete response rates remain low. Therefore additional treatment programs have been sought for initially unresectable cases. Recently, surgical treatments aiming at cure for patients with initially unresectable, but with good disease control, so-called “conversion surgery” have been frequently reported for several cancer types including esophageal and esophagogastric junction cancers [5, 6].

In this review, we will discuss the definition of conversion surgery for esophageal squamous cell carcinoma (ESCC) and esophagogastric junction adenocarcinoma (EGJAC), as well as safety and efficacy on patient survival. Good understanding of the existing evidence may contribute to the development of novel treatment strategy for initially unresectable disease, thus improve prognosis for patients with ESCC and EGJAC.

## Definition of conversion surgery

The concept of “conversion treatment” have been discussed well and established during gastric cancer (GC) treatment, and has been incorporated in ESCC and EGJAC. Conversion surgery for GC has been defined as a surgical treatment aiming at an R0 resection after chemotherapy for tumors that were originally unresectable or marginally resectable for technical and/or oncological reasons. In 2016, Yoshida et al. [5] proposed new categories of classification for stage IV GC, based on the absence and the presence of macroscopically detectable peritoneal dissemination and its potential resectability. The result of a large international multicenter retrospective study regarding conversion surgery for GC and EGJAC according to the proposed classification [7] was recently reported as discussed below.

Definition of conversion surgery for ESCC basically followed the decision for GC, except that ESCC has two unique features different from GC; first, is that ESCC often becomes unresectable due to adjacent organ invasion, and second, is that definitive chemoradiotherapy may be another option as conversion treatment. A multimodality treatment conference with experts from across East Asia was held to establish a consensus, and an agreement was reached that conversion therapy is defined as surgery or chemoradiotherapy aiming at cure after initial treatment for tumors that were initially unresectable due to adjacent organ invasion or distant metastasis [6].

**Fig. 1:**
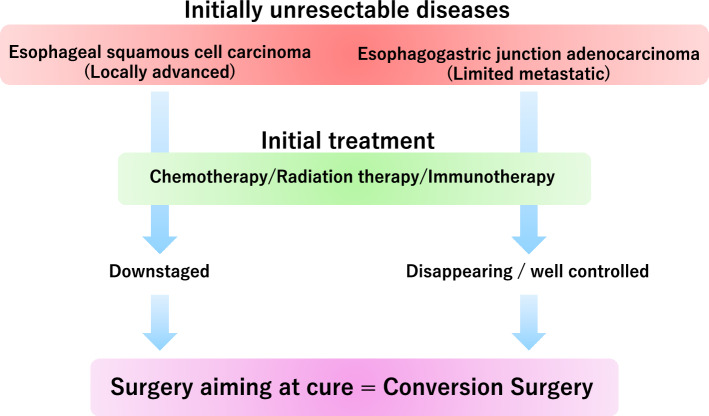
Conversion surgery for esophageal cancer and esophagogastric junction cancer

As above, conversion surgery for ESCC and EGJAC may be defined as surgical intervention for lesions that was originally unresectable due to oncological or technical reasons, which became resectable after downstaged by initial treatment (Fig. 1).

## Recent studies on conversion surgery

### Esophagogastric junction adenocarcinoma

CONVO-GC-1 [7] was a large retrospective international cohort study to clarify the short- and long-term outcomes of conversion therapy for stage IV GC, possibly including a certain number of patients with EGJAC. The primary endpoint was postoperative complication rates, and the secondary endpoint was overall survival (OS) according to the category classification as described above. A total of 1206 patients, 789 patients without peritoneal dissemination (categories one and two), and 417 patients with peritoneal dissemination (categories three and four), who underwent surgery after chemotherapy with curative intent were included in the analysis. Although the tumor location is unknown, certain numbers of EGJAC may be included in the 814 and 18 cases which underwent total and proximal gastrectomy, respectively. Significant postoperative complications classified as Clavien–Dindo (CD) grade IIIa or above occurred in 124 patients (10.3%), and overall fatal postoperative complication rate was 0.3%. The median survival time (MST) for the 1206 patients was 36.7 months (95% confidence interval [CI], 34.4–44.1). Although there was no obvious difference in survival outcomes between the four categories, patients who underwent R0 resection had prolonged MST (56.6 months, 95% CI; 46.4–74.5) compared to those who underwent R1 (25.8 months, 95% CI; 22.4–30.2) and R2 (21.7 months, 95% CI; 18.6–22.8) resection, respectively.

In a recent prospective multicenter phase II JCOG1704 trial [8] performed by the Japan Clinical Oncology Group (JCOG), utility of preoperative docetaxel, oxaliplatin, and S-1 (DOS) triplet therapy was assessed in GC patients with extensive LN metastasis. The primary endpoint was major pathological response rate. Four (9%) of the 47 patients included had esophageal involvement (which can be classified as EGJAC), and 27 (57%) patients had cM1 (para-aortic LN, PALN) diseases. Major pathological response rates and pathological complete response rates were 57%, and 24%, respectively. CD grade III or higher postoperative complications were pancreatic fistula (5%), abdominal abscess (2%), anastomotic leakage (2%), and pleural effusion (2%), and no in-hospital deaths were reported during the study.

A prospective phase II AIO-FLOT3 trial [9] included 252 patients with resectable or metastatic GC/EGJAC which were stratified into 3 groups: resectable, limited metastatic, and extensive metastatic diseases. Limited metastatic disease included cM1 disease with lymph node metastasis, one incurable organ- site metastasis, and localized peritoneal carcinomatosis either alone or in combined. Sixty patients with limited metastatic diseases underwent at least 4 cycles of preoperative fluorouracil, leucovorin, oxaliplatin, and docetaxel (FLOT), followed by surgical resection when curative resection was expected. MST for this cohort was 22.9 months (95% CI, 16.5—upper level not achieved), and MST for the 36 patients (60%) which proceeded to surgery (31.3 months; 95%CI, 18.9-upper level not achieved) was longer compared to the other patients (15.9 months; 95% CI, 7.1–22.9). Serious adverse events for the patients who underwent conversion surgery occurred in 3 patients (8.3%), which included anastomotic leak, pneumonia, and pleural complication.

As above, clinical trials on conversion surgery for EGJAC have been usually performed as part of studies for GC. Therefore, the safety and the utility of conversion surgery for EGJAC alone remain unknown. Nevertheless, the above recent clinical studies summarized in Table 1 suggest that conversion surgery for metastatic EGJAC can be safely performed, and prolonged survival may be achieved when R0 resection is accomplished. Since conversion surgery for metastatic EGJAC was limited to patients with extensive LN metastasis or single organ metastasis in recent prospective trials [8, 9], at present, indication for conversion surgery may need to follow these conditions. Phase III RENAISSANCE (AIO-FLOT5) trial [10] is now undergoing to verify the effect of chemotherapy followed by surgical resection versus chemotherapy alone on patient survival and quality of life for patients with limited metastatic GC / EGJAC. Further accumulation of cases is needed to decide the utility of conversion surgery in each organ metastasis.

**Table 1:** Recent studies on conversion surgery for gastric cancer and esophagogastric junction adenocarcinoma included in this review

Author	Year	Study design	Cancer type	Disease stage	Preoperative therapy	N	R0 rate	Clavien-dindo grade 3 or above postoperative complications						MST (months)	
								All	Anastomotic leakage	Abdominal abscess	Pancreatic fistula	Pneumonia	Death	ALL	R0
Yoshida et al. [7]	2021	Retrospective	GC	Stage IV	Multiple	1206	69.6%	10%	3%	4%	5%	2%	0.2%	36.7	56.6
Kurokawa et al. [8]	2024	Phase II	GC	Bulky nodal involvement or PALN metastasis	DOS	47	93%	11%	2%	2%	5%	0	0	–	–
Al-Batran et al. [9]	2017	Phase II	GC/EGJAC	Limited metastatic	FLOT	67	54%	8%	35	0	0	3%	0	22.9	31.3

*GC* gastric cancer, *EGJAC* esophagogastric junction adenocarcinomar, *PALN* para-aortic lymph node, *DOS* docetaxel+oxaliplatin+S-1, *FLOT* 5-fluorouracil+leucovorin+oxaliplatin+docetaxel, *MST* median survival time

## Esophageal squamous cell carcinoma

### Locally advanced diseases

Surgical strategy for locally advanced unresectable ESCC includes conversion surgery after chemoradiotherapy (CRT) or induction chemotherapy (ICT). Studies on conversion surgery after CRT, so-called “salvage surgery” often reported a prolonged survival for patients who achieved R0 resection compared to non-curative cases, although, surgery was related to high morbidity, such as anastomotic leakage of up to 38% and respiratory complication of up to 62%, and mortality of up to 10% [11–16]. Recently, more intensive chemotherapeutic regimen such as triplet chemotherapy has been developed, and has been introduced into initially unresectable cases as ICT. In a multicenter phase II study in Japan [17], safety and efficacy of docetaxel, cisplatin, and 5-fluorouracil (DCF) triplet ICT followed by conversion surgery were investigated in patients with initially unresectable locally advanced ESCC. The primary endpoint was 1-year OS. Within the enrolled 48 patients, conversion surgery was performed in 20 (41.7%) patients, and R0 resection was confirmed in 19 (39.6%) patients. The estimated 1-year OS was 67.9%, with an acceptable safety profile, in which no serious postoperative complications were observed. In the long-term analysis [18], the estimated 3-year OS was 46.6% (95% CI, 34.2–63.5%), and the 3-year OS and the progression-free survival (PFS) were significantly longer for the patients who underwent R0 resection (n = 19) than for those who did not (3-year OS, R0; 71.4%, non-R0; 30.1%, (3-year PFS, R0; 61.3%, non-R0; 25.0%), respectively.

A recent multicenter randomized phase II trial compared CRT versus DCF-ICT as initial induction therapy for unresectable locally advanced ESCC [19, 20]. A total of 99 patients were either randomized to induction CRT or DCF-ICT, and conversion surgery was performed if considered resectable after initial or secondary treatment. The primary endpoint was 2-year OS. Although R0 resection rate (CRT, 78%; DCF-ICT, 76%, *P* = 1.000) and the overall incidence of postoperative complications (CRT, 55%; DCF-ICT, 59%, *P* = 0.824) were similar between the groups, adverse events during the initial treatment were more frequently observed in patients after DCF-ICT, and patients after CRT achieved better histological complete response rate (CRT, 40%; DCF-ICT, 17%, *P* = 0.028). In the final analysis, although not significantly different, 2-year OS was relatively higher after CRT compared to DCF-ICT (CRT, 55.1%; DCF-ICT, 34.7%, *P* = 0.11), suggesting that CRT may be another option as an induction treatment for unresectable locally advanced diseases.

Recent studies summarized in Table 2 have indicated that regardless of the preoperative treatment, conversion surgery for initially unresectable locally advanced ESCC can be safely performed [21]. However, its survival benefit remains unclear. A randomized phase III trial to confirm the superiority of ICT-DCF followed by conversion surgery or definitive CRT over definitive CRT alone for OS in patients with locally advanced unresectable ESCC by the JCOG (JCOG1510) [22] was commenced in 2018 and finished patient recruitment. Long-term survival outcome is awaited to demonstrate the prognostic benefit of conversion treatment.

**Table 2:** Prospective studies on conversion surgery for locally advanced esophageal squamous cell carcinoma

No.	Author	Year	Study design	N	Initial treatment	CS rate	R0 rate	Postoperative complications			Overall survival rates			
								RLNP	Leakage	Pneumonia	1-year	2-years	3-years	5-years
1	Ikeda et al. [11]	2001	Phase II	37	CF-RT	35.1%	32%	–	–	35%	45%		–	23%
2	Fujita et al. [15]	2005	Cohort	26	CF-RT	58%	42％	50%	21%	43%	65%		23%	19%
3	Shimoji et al. [16]	2013	Cohort	43	FAN, 5-FU+NDP+RT, DTX+RT	70%	61%	17%	13%	47%	–		–	35%
4	Yokota et al. [17, 18]	2016, 2020	Phase II	48	DCF	42%	40％	38％	–	14％	66.7%		46.6%	–
5	Sugimura et al. [19, 20]	2021, 2023	Phase II	49	CF-RT	84%	78%	18%	13%	18%	–	55.1%	–	–
				50	DCF	84%	76%	12%	10%	37%	–	34.7%	–	–

CF-RT, Cisplatin+5-fluorouracil+radiation therapy; FAN, 5-fluorouracil+doxorubicin+nedaplatin; 5-FU, 5-fluorouracil; DTX, Docetaxel; DCF, Docetaxel+Cisplatin+5-fluorouracil; CS, conversion surgery; RLNP, recurrent laryngeal nerve paralysis.

### Metastatic diseases

Possibly due to the development of more effective treatment, conversion surgery for metastatic ESCC has started to be reported recently. In a single-center retrospective study which included 13 ESCC patients with solitary abdominal PALN metastasis who underwent conversion surgery [23], 3 patients had pathologically positive PALN, and induction treatment eliminated cancer cells in PALN in 6 patients among 10 patients with negative PALN. Three (23.1%) patients had grade II or higher postoperative complications, and the 3-year OS and RFS rates were 83.1% and 51.3%, respectively. In another retrospective study [24], 80 ESCC/EGJAC patients with potentially resectable M1 LN metastasis including supraclavicular, pretracheal, posterior thoracic para-aortic, and abdominal para-aortic LN metastasis without organ metastasis underwent preoperative DCF or FP therapy followed by surgical treatment. There were no significant differences between patients with or without M1 LN in terms of short-term safety outcomes and OS.

In a multi-institutional retrospective study reported in 2024 [25], 66 patients with metastatic ESCC, including 51 and 15 patients with synchronous distant LN or organ metastasis, respectively, underwent induction chemo(radio)therapy followed by surgery. Within 66 patients, 65 patients were diagnosed as having ESCC histologically. During the initial treatment, DCF triplet chemotherapy, CF therapy, and chemoradiotherapy were performed in 53 (80%), 10 (15%), and 3 (5%) patients, respectively. R0 surgery was achieved in 61 (92%) patients. Postoperative complication occurred in 31 (47%) of the patients, in which pulmonary complication (20%), anastomotic leakage (9%), and recurrent laryngeal nerve palsy (8%) being the frequent complications. In-hospital death occurred in 1 patient, and the 3- and 5- year OS rates were 32.4% and 24.4%, respectively. In this study, the OS rates were similar between patients with distant LN metastasis and organ metastasis (3-year OS, distant LN metastasis; 34.9%, distant organ metastasis; 26.7%, *P* = 0.435).

Recent evidence suggests that conversion surgery for ESCC with potentially resectable distant LN metastasis may be an effective treatment strategy with acceptable safety profile, although prospective studies are lacking. Further research is needed to verify the utility of conversion surgery against organ metastasis.

## Best timing for conversion surgery

Although further accumulation of evidence is inevitable, previous studies indicate that conversion surgery for ESCC and EGJAC may be safely performed, and survival benefit may be achieved when R0 resection is accomplished as discussed above. The best timing for conversion surgery should be when the primary/metastatic lesion represents the best response to preoperative treatment [5], although currently, there is no established method to predict best response. Timing of surgery was different among studies included in this review; however, it was typically performed after 1–2 courses of CRT or 2–3 courses of ICT for locally advanced diseases (Table 2), and 3–4 courses of ICT for metastatic diseases (Table 1), which seems similar to neoadjuvant settings. Longer treatment may improve R0 resection rate especially for locally advanced diseases. However, on the other hand, we may lose a chance to perform conversion surgery for tumors with treatment resistance. Close follow-up by various imaging inspections such as endoscopy, CT, and MRI may be needed during the induction treatment for the early detection of tumor regrowth, in order to provide conversion surgery as a treatment option for the patient. Another important topic is the safety of conversion surgery at different time points. Since the safety of surgery after prolonged preoperative treatment has not yet been clarified, treatment schedule similar to neoadjuvant settings, in which the safety has been indicated by various clinical trials [26–28], might be a safe option at present. Finally, as discussed below, introduction of ICI may lead to better chance of conversion surgery. However, the best timing of surgery after ICI-based induction therapy needs to be further discussed.

## Future of conversion surgery: induction immunotherapy

Development of a more intensive induction treatment may improve R0 resection rate, thus lead to better survival outcomes if the safety is guaranteed. Recently, ICI + chemotherapy or doublet ICI has been approved for the treatment of ESCC and EGJAC in recurrent/metastatic settings [1–4, 29], and shown better response and equivalent safety profile compared to previous chemotherapeutic treatment, thus resulting in prolonged survival outcomes. Further, studies on perioperative ICI therapy in patients with resectable ESCC and EGJAC [30–32] suggests that perioperative ICI and the following surgical treatment may be safely performed, offering a promising response. These results raise a question if induction ICI contribute to improved survival outcomes for patients with initially unresectable ESCC/EGJAC.

In a retrospective multicenter study in China, [33], 155 patients with advanced ESCC, including 102 (65.8%) patients with locally advanced initially unresectable disease underwent induction ICI + chemotherapy. The following surgical resection was offered to 116 patients (74.8%), with a R0 resection rate of 94%. The objective response rate was significantly higher in the ICI + chemotherapy cohort compared to an independent cohort receiving induction chemotherapy alone (ICI + chemotherapy, 63.2%; chemotherapy, 47.7%; P = 0.004). Patients who underwent conversion surgery showed significantly higher event-free survival compared to those who did not. Regarding safety analysis, 107 (69.0%) of the patients experienced at least one treatment-related adverse events, including 45 (29.0%) patients with grade 3 or higher adverse events. In another single-center retrospective cohort study in China [34], 136 patients with stage IV GC, including 30 (22.1%) patients with upper-third GC underwent combination of ICI + chemotherapy or HER 2 targeted therapy. Among the 42 patients who underwent the following conversion surgery, R0 resection was performed for 38 (90.5%) patients, and OS and PFS were 96.6% and 89.1%, respectively. No serious complications leading to death were reported during the perioperative period. In this study, programmed death ligand1combined positive score (CPS) ≧5 (odds ratio, 0.22; 95% CI, 0.08–0.57; *P* = 0.002) favored successful conversion surgery.

At our institute, 14 patients with initially unresectable ESCC/EGJAC underwent ICI based treatment followed by conversion surgery, with a follow-up period of longer than one year (Table 3). All patients underwent successful R0 resection.　Pathological complete response was seen in 2 (14.3%) patients, and the patients with pathological response ≧ Grade 2 had favorable survival outcomes without recurrence. One-year OS and RFS rates were 85.7% and 78.6%, respectively, which seems promising for Stage IV diseases. However, immune-related adverse events (iRAEs), such as hypoadrenalism and interstitial pneumonia, were observed during induction therapy (28.6%) and postoperative period (21.4%), which should be carefully managed in order to avoid deterioration and discontinuation of treatment.

**Table 3:** Conversion surgery for esophageal/esophagogastric junction cancer after immune check point inhibitor immunotherapy at our institute

No.	Cancer type	Unresectable factor	Induction therapy	Courses	iRAEs	Pathological response	Postoperative iRAEs	Recurrence
1	EGJAC	Distant LN	ICI+chemotherapy	4	Hypoadrenalism	2	–	–
2	ESCC	Lung	ICI+chemotherapy	11	–	1a	Fulminant type 1 diabetes	–
3	EGJAC	Distant LN	ICI+chemotherapy	5	–	1a	–	–
4	ESCC	Distant LN	ICI doublet	3	–	2	–	–
5	ESCC	Distant LN	ICI+chemotherapy	3	Hypopituitarism	2	Interstitial pneumonia	–
6	ESCC	Distant LN	ICI+chemotherapy	3	–	1b	–	+
7	ESCC	T4b	ICI+chemotherapy	3	–	2	–	–
8	EGJAC	Distant LN	ICI+chemotherapy	4	Hypothyroidism	2	Interstitial pneumonia	–
9	EGJAC	Peritoneal	ICI+chemotherapy	5	–	1a	–	+
10	ESCC	Skin	ICI+chemotherapy	7	–	1b	–	+
11	ESCC	Distant LN	ICI+chemotherapy	3	–	3	–	–
12	ESCC	T4b	ICI+chemotherapy	3	–	2	–	–
13	EGJAC	Distant LN	ICI doublet	7	Hypoadrenalism	3	–	–
14	ESCC	Distant LN	ICI+chemotherapy	6	–	1a	–	–

Pathological response is based on Japanese Classification of Esophageal Cancer, 12th Edition

*ESCC* esopageal squamous cell carcinoma, *EGJAC* esophagogastric junction adenocarcinoma, *LN* lymph node, *ICI* immune checkpoint inhibitor immunotherapy, *iRAEs* immune-related adverse events

As thus far, there seems to be only case reports and small-scale retrospective studies. However, ICI-based induction therapy may provide novel treatment strategies for initially unresectable diseases. CPS and pathological response may be surrogate endpoints for survival outcomes. Future prospective studies, or alternatively, multi-institutional large retrospective studies are awaited to demonstrate the safety and utility of induction ICI + conversion surgery. Early diagnosis and initiation of treatment for iRAEs are essential to minimize patient disadvantages.

## Conclusions

Recent development of the induction treatment has enabled more patients to undergo conversion surgery, and thus improved survival for initially unresectable ESCC/EGJAC. Improvement of the postoperative management has ensured surgical safety, and also contributed to improved outcomes. Novel immunotherapeutic strategies have high expectations to further improve prognosis for the patients. However, measures against specific complications are required. Although multiple issues, such as optimal induction regimen, surgical approach, and correct timing for surgical intervention remain unclear, conversion surgery seems to be an essential approach to improve outcomes for the patients with initially unresectable diseases. Results of the ongoing and future clinical trials are awaited.

## Data availablility

All data which support the findings of this study are included within this paper.
